# Function and Therapeutic Implications of tRNA Derived Small RNAs

**DOI:** 10.3389/fmolb.2022.888424

**Published:** 2022-04-13

**Authors:** Briana Wilson, Anindya Dutta

**Affiliations:** ^1^ Department of Biochemistry and Molecular Genetics, University of Virginia School of Medicine, Charlottesville, VA, United States; ^2^ Department of Genetics, University of Alabama, Birmingham, AL, United States

**Keywords:** tRNA fragments, RNA therapeutics, translation inhibition, post-transcriptional regulation of gene expression, RNA silencing

## Abstract

tRNA derived small RNAs are mainly composed of tRNA fragments (tRFs) and tRNA halves (tiRs). Several functions have been attributed to tRFs and tiRs since their initial characterizations, spanning all aspects of regulation of the Central Dogma: from nascent RNA silencing, to post-transcriptional gene silencing, and finally, to translational regulation. The length distribution, sequence diversity, and multifaceted functions of tRFs and tiRs positions them as attractive new models for small RNA therapeutics. In this review, we will discuss the principles of tRF biogenesis and function in order to highlight their therapeutic potential.

## Introduction

The widespread introduction of small RNA sequencing technologies has uncovered a wide variety of non-microRNA (miRNA) small RNAs (nmsRNA) (Lee et al., 2009; Falaleeva and Stamm, 2013; Jackowiak et al., 2017; Cherlin et al., 2020). tRNA fragments (tRFs) and tRNA halves (tiRs) are one of the most highly abundant class of small RNAs, and depending on the cell type or condition, may reach higher levels than miRNAs (Sharma et al., 2016). Initially thought to be degradation products of tRNAs, the smaller tRFs were systematically characterized and found to have discrete length peaks (Kumar et al., 2014). Later, the longer tiRs were discovered and were found to be the result of tRNA cleavage by angiogenin (ANG) during stress (Yamasaki et al., 2009).

Initial characterizations focused primarily on tRFs having a miRNA-like mechanism, due to the similar size distribution between miRNAs and tRFs (Lee et al., 2009; Haussecker et al., 2010). To date, however, several other functions have been identified, covering all aspects of the Central Dogma including nascent RNA silencing, post-transcriptional gene silencing, and translational regulation (Yamasaki et al., 2009; Haussecker et al., 2010; Guzzi et al., 2018; Kuscu et al., 2018; Fricker et al., 2019; Di Fazio et al., 2022). While tRFs and tiRs have experienced a boom in basic biological and functional (Table 1) insights, miRNAs and siRNAs have experienced a renaissance in their therapeutic applications. Although many reviews focus on the role of tRFs and tiRs in disease and as potential biomarkers (Jia et al., 2020; Zeng et al., 2020; Fagan et al., 2021; Zong et al., 2021), here, we will discuss recent tRF and tiR functional insights, with emphasis on their potential therapeutic applications.

**TABLE 1:** The multifaceted functions of tRFs and tiRs.

Function	tRF type	tRF Examples	References
Post-transcriptional gene silencing (*via* association with RISC)	tRF-5s	several tRF-5s (bind AGO1,3,4)	Kumar et al. (2014)
	tRF-3s	several tRF-5s (bind AGO1,3,4)	Kumar et al. (2014)
	tRF-3	tRF-3001a,tRF-3003a, tRF-3009a	Kuscu et al. (2018)
	tRF-3	cand14	Haussecker et al. (2010)
	tRF-3	CU1276	Maute et al. (2013)
	tRF-3	Bj-tRF001,Bj-tRF002	Ren et al. (2019)
	tRF-5	Bj-tRF003	Ren et al. (2019)
	tRF-5	tRF5-GluCTC	Deng et al. (2015)
Role in cell proliferation	tRF-1	tRF-1001	Lee et al. (2009)
Endogenous retroviral reverse transcriptional silencing	tRF-3	tRF ETn (18 nt) (MERV)	Schorn et al. (2017)
Exogenous retroviral reverse transcriptional silencing	tRF-3	PBSncRNA (HIV)	Yeung et al. (2009)
Exogenous retroviral reverse transcriptional enhancement	tRF-3	tRF-3019 (HTLV-1)	Ruggero et al. (2014)
Endogenous retroviral post-transcriptional silencing	tRF-3	tRF ETn (22 nt) (MERV)	Schorn et al. (2017)
Endogenous retroviral chromatin mediated silencing	tRF-5	tRF-GlyGCC (MERVL)	Sharma et al. (2018), Boskovic et al. (2020)
Nascent RNA silencing	tRF-3 (precursor)	tsRNA SPINT1	Di Fazio et al. (2022)
	tRF-5	tsRNA LINC00665	Di Fazio et al. (2022)
	tRF-5	tsRNA EGFR/MET	Di Fazio et al. (2022)
	i-tRF	tsRNA BCL2	Di Fazio et al. (2022)
Translational gene silencing	tRF-5	Val-tRF	Gebetsberger et al. (2012)
	tRF-5	tRF(Gln)	Sobala and Hutvagner (2013)
	tRF-5	tRF(Val)	Sobala and Hutvagner (2013)
	tRF-5	tRF(Lys)	Sobala and Hutvagner (2013)
	tiRs	stress-induced tiRs	Yamasaki et al. (2009)
	tiR-5	tRNA-Ala	Ivanov et al. (2011)
	tiR-5	tRNA-GlyGCC	Ivanov et al. (2011)
	tiR-5	tRNA-GlyCCC	Ivanov et al. (2011)
	tiR-5	tRNA-Cys	Ivanov et al. (2011)
	tiR-3	tRNA-Pro	Ivanov et al. (2011)
Translational enhancement	tiR-3	tRNA-ThrAGU (*T. brucei*)	Fricker et al. (2019)
	tRF-3	tRNA-LeuCAG	Kim et al. (2017)
RNA protein binding	i-tRFs	tRNA-Glu,-Asp,-Tyr,-Gly (binds YBX1)	Goodarzi et al. (2015)
	tRF-3	tRNA-Glu (binds NCL)	Falconi et al. (2019)
	tiRs	20 ANG-dependent tiRs (binds Cytochrome C)	Saikia et al. (2014)
	tRF-1	tRF_U3_1 (binds SSB/La)	Cho et al. (2019)

## Biogenesis of tRNA Fragments and tRNA Halves

**FIGURE 1:**
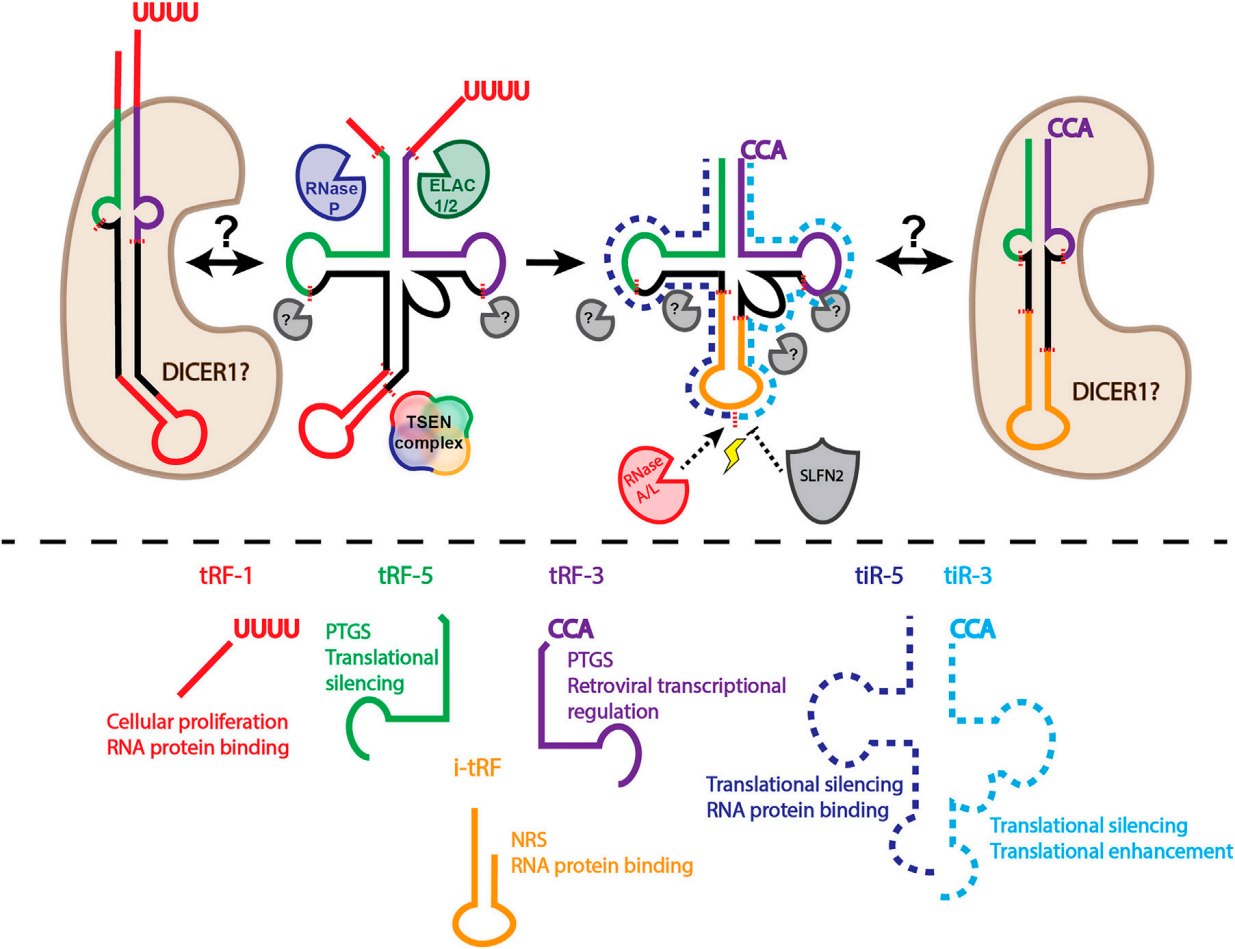
Biogenesis and classification of tRFs and tRNA halves. Above dashed line: tRNAs undergo extensive processing and modification to generate a mature tRNA capable of being amino acylated. Precursor tRNAs have 5′ leader sequences removed by RNase P. 3′ trailer sequences removed by ELAC1/2 (also known as RNase Z) produces tRF-1s. Introns must be removed by the TSEN complex. Addition of a non-templated CCA, along with installment of necessary RNA modifications completes tRNA maturation. Cleavage of the mature tRNA produces tRFs classified based on length and origin on the parental tRNA. It is unclear if DICER1 is responsible for tRF biogenesis when tRNAs are folded in an alternative hairpin structure, rather than the canonical cloverleaf structure. It is important to note that DICER1 knockout does not eliminate production of many tRFs, leaving the identity of the enzyme that is necessary for tRF biogenesis unknown. In stressful conditions (lightning bolt), RNase A family members, such as ANG, cleave mature tRNAs in the anticodon loop, producing tRNA halves (tiRs). SLFN2 can block ANG mediated tRNA cleavage under stress conditions. Below dashed line: classification of tRFs and tiRs and the top two functions associated with each class based on the amount of experimental evidence. The functions also represent the potential therapeutic application for each tRF/tiR class. PTGS, post-transcriptional gene silencing; NRS, nascent RNA silencing.

tRNA derived small RNAs are divided into two major classes based on length: shorter tRFs and longer tRNA halves (Figure 1). The two major classes can be further subdivided based on the location from which they arise on the parental tRNA. tRNA halves are 31–40 nucleotides long and generally arise from a mature parental tRNA (Kumar et al., 2016; Su et al., 2020). tRNA halves are classified based on whether it comes from the 5′ or 3′ end (tiR-5s come from the 5′ end, tiR-3s from the 3′ end). tRFs are generally between 14 and 30 nucleotides. tRF-5s come from the 5′ end of the mature parental tRNA. tRF-3s arise from the mature parental 3′ end; tRF-1s come from the trailer of the parental precursor tRNA. tRF-5s and tRF-3s can be further subclassified based on length distribution, with peaks of tRF-5s at 14 to 16 nucleotides (tRF-5a), 22 to 24 nucleotides (tRF-5b), and 28 to 30 nucleotides (tRF-5c). tRF-3s have peak lengths at around 18 nucleotides (tRF-3a) and 22 nucleotides (tRF-3b) (Kumar et al., 2016; Su et al., 2020).

The discrete length distributions of tRFs suggest specific cleavage by cellular ribonucleases. However, with the exception of tRF-1s, a general biogenesis mechanism for highly expressed tRFs remains to be elucidated. tRF-1s were one of the first tRFs to be characterized (Lee et al., 2009). tRF-1s arise from RNase Z (also known as ELAC1/2) cleavage of the precursor tRNA trailer sequence (Figure 1). tRF-5s and tRF-3s on the other hand, have a generally unelucidated biogenesis mechanism (Figure 1). Several groups have suggested the role of DICER1 in tRF biogenesis (Maute et al., 2013; Liu S. et al., 2018; Reinsborough et al., 2019; Di Fazio et al., 2022), however DICER1 knockout cell lines still have unaltered amounts of several highly abundant tRF-5s, tRF-3s, and tRF-1s (Kumar et al., 2016; Kuscu et al., 2018). Although DICER1 may not play a general role in tRF biogenesis, some tRFs may be produced by DICER1 cleavage. For example, Hasler et al. (2016) found that in the absence of the tRNA maturation protein SSB/La, some tRNAs may fold into an alternative structure, producing a hairpin (Hasler et al., 2016). This hairpin structure is then an optimal substrate for DICER1 cleavage. Additionally, Di Fazio et al. (2022) show that a subset of tRFs decrease under DICER1 knockdown conditions, and that tRNAs incubated with DICER1 can produce tRFs (Di Fazio et al., 2022). The discrepancy between Di Fazio et al. (2022) and Kumar et al. (2016) or Kuscu et al. (2018) may be due to the tRFs analyzed. Perhaps the different approaches for DICER1 depletion also affect the levels of tRFs. One technical limitation of both studies is that no spike-ins were used. Since miRNAs make up the majority of reads in small RNA sequencing data, and total mapped reads are used to normalize small RNA sequencing data, a substantial amount of bias can be introduced. Finally, a DICER1 dependent and DICER1 independent biogenesis mechanism are not incompatible. Perhaps, as Hasler et al. (2016) have shown for a particular subset of tRFs, some tRNAs can fold into a hairpin structure, making them a higher affinity DICER1 substrate, while other tRNAs are more likely to form a canonical cloverleaf structure (Figure 1). The propensity of a particular tRNA to fold into a hairpin vs. cloverleaf and how this influences tRF biogenesis is understudied. However, it would not be surprising if there were cell type variations due to differential tRNA expression, modification, or protein binding. As such, these experiments would require a modification-aware tRNA folding approach.

tiR biogenesis may also have more than one mechanism. Initially, tiRs were found to be upregulated under various stress conditions, giving them the name stress-induced tRNA halves. The RNase IV enzyme, angiogenin (ANG), was identified to be the enzyme responsible for tiR production under stress conditions and the 5′-terminal oligoguanylate (TOG) motif was found to be present in these tiRs (Ivanov et al., 2011; Saikia et al., 2014; Honda et al., 2015; Liu S. et al., 2018; Hogg et al., 2020). However, ANG KO does not reduce the levels of basally expressed (non-stress induced) tiRs (Su et al., 2019). Evidence of tiR production by other RNases is accumulating. RNase L was found to produce tiRs in a TOG independent manner (Donovan et al., 2017). Other RNase A family members have also been suggested to play a role in tiR production (Akiyama et al., 2019). These findings highlight the granularity of tRF and tiR production: ultimately, the identification of a grand, unifying biogenesis mechanism for all tRFs and tiRs may not be attainable.

## Function of tRFs and tiRs

### tRFs Enter RISC and Engage in Post-transcriptional Gene Silencing

In order to highlight the potential of tRFs to enhance therapeutic small RNA development, it is important to understand their endogenous cellular roles. The first report of a biological function for tRFs was reported for tRF-1001 (Lee et al., 2009). This tRF was highly abundant in cancer cell lines and was found to be required for cell proliferation. Of note, no detectable miRNA-like function was found for tRF-1001 in this study. The mechanism by which tRF-1001 regulates cellular proliferation still remains to be determined, but a miRNA-like mechanism is unlikely.

The lack of miRNA-like functions for tRF-1s was also established in another study (Haussecker et al., 2010). Transfection of a reporter and an oligonucleotide antisense to cand45 (tRFdb ID tRF-1001) did not derepress the luciferase reporter. Surprisingly, the reporter was further repressed by anti-tRF-1001 introduction. These results may be explained by a mechanism in which addition of an antisense oligo stabilizes tRF-1001, rather than interfering with its function. Interestingly, a more global meta-analysis of tRFs associated with AGO proteins revealed a striking absence of tRF-1s with AGO1-4 (Kumar et al., 2014). Both results make it clear that tRF-1s generally lack base-pair mediated repression of target mRNAs under basal conditions.

In the one case that tRF-1s are associated with miRNA-like repression, the tRF-1 is generated by DICER1 cleavage. DICER1 cleavage of tRNAs can occur when the precursor tRNA maturation protein, SSB/La, is depleted (Hasler et al., 2016). Under these conditions, RNase Z cannot cleave the precursor trailer sequence, allowing the tRNA to possibly form a hairpin, a substrate amenable to DICER1 cleavage. Overexpression of the tRF-1, either *via* mimic transfection, shRNA, or parental tRNA mediated overexpression led to repression of a luciferase reporter. Although these data clearly support a role for tRF-1 mediated repression, it is unclear if the effects are mediated by the SSB/La-DICER1 regulatory axis, as the parental tRNA overexpression followed by luciferase reporter was not conducted in SSB/La or DICER1 deficient cells.

Unlike tRF-1s, tRF-3s have been shown to readily enter RISC and repress gene expression in a base-pairing specific manner. As alluded to previously, overexpression of parental tRNAs leads to a direct upregulation of tRF expression. Parental tRNA overexpression leads to tRF-3001a, -3003a, and -3009a generation and entry into AGO for efficient repression of luciferase reporters (Kuscu et al., 2018). Haussecker et al. (2010) also showed that type I tRFs (tRF-3s) can behave like miRNAs because transfection of an antisense oligo derepressed a luciferase reporter. In the context of cancer, CU1276 (tRF-3027b) was found to be absent from germinal center derived lymphomas, present in normal germinal centers, and can downregulate RPA1 in a sequence specific manner (Maute et al., 2013). For tRF-3 the evidence is strong that the repression does not require DICER1, because tRNA overexpression mediated repression of tRF-3 reporters continues in DICER1 knock-out cells (Kuscu et al., 2018). Importantly, the repression of tRF-3 targets when tRNAs are overexpressed requires AGO and requires seed-match with the 3’ UTR of the target (Kuscu et al., 2018).

The difference between the ability of tRF-1s and tRF-3s to repress target gene expression is unclear. The answer may lie in the inherent differences between the two classes: 1) different biogenesis enzymes (Vogel et al., 2005); 2) tRF-1s are likely to be single stranded (Lee et al., 2009), while the biogenesis of tRF-3s may involve a double stranded intermediate; or 3) undescribed sequence or modification difference; 4) differential association with RISC accessory proteins (Haussecker et al., 2010).

Another report found that two tRF-3s and one tRF-5 produced by rhizobial bacteria are enriched in soybean root nodules (Ren et al., 2019). These tRFs appeared to regulate soybean host genes in a miRNA-like manner because rhizobial tRF targets were predicted using plant miRNA target rules, overexpression of mimic rhizobial tRFs repressed predicted targets, and depletion of rhizobial tRFs resulted in an increase in predicted targets. A tRF-5 was also identified to regulate gene expression post-transcriptionally *via* base-pairing in the context of respiratory syncytial virus (RSV) infection (Deng et al., 2015). In this case, a tRF-5 derived from GluCTC downregulated expression of APOER2, which enhanced RSV replication. These findings highlight the ability of tRFs to behave like bona fide miRNAs, function in viral pathogenesis, and function across the kingdoms of life.

Perhaps one of the most important roles for tRFs may be in the function of stem cells and germ cells, hinting at a fundamental and primordial function for this class of small RNAs derived from tRNAs. tRFs have been found to be highly expressed in mouse stem cells where they suppress retrotransposition of endogenous retroviruses (Schorn et al., 2017). The 18 nucleotide tRF-3as accomplish this by blocking reverse transcription. 22 nucleotide tRF-3bs suppress retrotransposition *via* post-transcriptional silencing of retrotransposon gene expression. tRF’s ability to interact with a retroelement may not come as a surprise, since retroviruses usurp host cell tRNAs for priming in reverse transcription (Mak and Kleiman, 1997). In another example of tRF’s roles in early embryogenesis and germ cells, tRFs were found to be highly abundant in mature mouse sperm (Sharma et al., 2016; Sharma et al., 2018). tRF-5 from tRNA-GlyGCC (tRF-GG) was determined to be a repressor of MERVL *via* regulation of histone protein level and Cajal-body dependent noncoding RNAs (Boskovic et al., 2020). Finally, it has been shown that tRFs derived from tRNA primers can inhibit HIV reverse transcription (Yeung et al., 2009), or enhance HTLV-1 reverse transcription (Ruggero et al., 2014).

### Therapeutic Potential of tRFs in Post-transcriptional Gene Silencing

Interestingly, most studies that determine tRF mediated repression, either endogenously or *via* tRNA or tRF mimic overexpression, observe at most a 40–60% reduction in target luciferase reporter expression (Haussecker et al., 2010; Maute et al., 2013; Kuscu et al., 2018). This is perhaps due to the fact that most tRFs appear to enter AGO1, 3 and 4, rather than AGO2 which has slicer activity. Overexpression of either AGO2 (Haussecker et al., 2010; Maute et al., 2013) or knockout of DICER1 (Kuscu et al., 2018) in these systems leads to enhanced repression. These data suggest that availability of AGO2 binding is essential for miRNA-like efficiency in repression. This makes potential tRF-like therapeutics less useful if maximal repression of the target gene is required, which may lead to questioning of the utility of tRF mediated post-transcriptional gene silencing as a therapeutic approach. However, the moderate attenuation of gene expression mediated by tRFs may prove useful in disease contexts in which gene expression needs to be knocked down, but some degree of expression is still required for normal function. For example, Beckwith-Wiedemann syndrome is an imprinting disorder in which IGF2, among other genes, expression is too high (Wang et al., 2019; Borjas Mendoza and Mendez, 2021). IGF2 is a necessary gene for intrauterine growth and development, so too much repression of this gene would be detrimental. In fact, loss of IGF2 expression leads to Russell-Silver syndrome, a congenital growth syndrome which leads to failure to thrive (Saal et al., 2002). Thus, the correct balance of IGF2 expression needs to be achieved, and a tRF-like mechanism may be more amenable to this sort of repression, rather than siRNA or miRNA-like approaches.

### tRFs can Regulate Nascent RNA Expression

One of the newest functions of tRFs is in nascent RNA silencing (Di Fazio et al., 2022). DICER1 dependent tRFs were found to target introns of genes in the nucleus in an AGO2 dependent manner. Nuclear functions of RISC have been reported (Gagnon et al., 2014), however, this is the first time tRFs have been attributed with this function. One potential reason tRFs may be uniquely suited to regulate nuclear gene expression either co- or post-transcriptionally may be their single stranded nature. David Corey and colleagues reported that single stranded small RNAs can readily bind nuclear AGO2, whereas double stranded siRNAs can only bind cytoplasmic AGO2, likely due to the nucleus missing accessory double stranded RNA loading factors.

There may be several reasons that tRFs are able to engage in nascent RNA silencing besides their single strandedness. One underexplored feature may be the modifications present on tRFs. It is possible that certain modifications allow tRFs to enter the nucleus and regulate gene expression. Although exciting, a more thorough understanding of nascent RNA silencing machinery and biochemistry will be necessary if it is to become a potential therapeutic modality.

### tRFs and tiRs can Alter Translation

tRF-3s, and under certain conditions, tRF-1s, have been shown to regulate gene expression post-transcriptionally *via* base-pairing (Haussecker et al., 2010; Maute et al., 2013; Deng et al., 2015; Kuscu et al., 2018; Ren et al., 2019). Sobala and Hutvagner suggested that tRF-5s do not function in this way, and in fact are capable of reducing mRNA translation in the absence of base-pairing (Sobala and Hutvagner, 2013). In particular, a “GG” dinucleotide appears to be necessary, but not sufficient, to mediate repression of a luciferase reporter (Sobala and Hutvagner, 2013). These tRFs repress translation by associating with polysomes. This work is in line with previous work showing that tRNA fragments and halves derived from the 5′ end of tRNAs generally inhibit translation across the domains of life (Yamasaki et al., 2009; Zhang et al., 2009; Ivanov et al., 2011; Gebetsberger et al., 2012). tiR-5s can be produced under stress conditions. For example, stress induced by arsenite treatment, heat shock, and UV radiation induced cleavage of tRNAs *via* ANG, which led to translation inhibition in a phospho-eIF2⍺ independent manner (Yamasaki et al., 2009). In a follow-up study, tiRs were found to work with YB-1 to displace eIF4G/A and eIF4F from uncapped mRNAs and m7G caps, respectively (Ivanov et al., 2011). Four to five guanines, or 5′ terminal oligo guanine (5′-TOG) motifs, were also found to be important for translation inhibition in this study, as tiR-5s from tRNA-Ala and tRNA-Cys with 5′-TOG motifs inhibit translation much more efficiently than tiRs without the motif. In sum, tiR-5s and tRF-5s seem to reduce global translation, and multiple guanines seem to be important for this mechanism.

As opposed to tiR-5s, much less seems to be known about tiR-3 function. A tiR-3 derived from tRNA-ThrAGU was found to be upregulated in starvation conditions in *Trypanosoma brucei*, one of the parasitic protozoans that causes the neglected disease sleeping sickness (Fricker et al., 2019). In contrast to tRF-5s and tiR-5s, the tiR-3 stimulated translation during starvation recovery (Fricker et al., 2019). Perhaps targeting this tiR-3 and preventing translation stimulation in post-starvation protozoa could be an alternative therapeutic approach for sleeping sickness caused by this subspecies.

### Therapeutic Potential of tRF and tiR Translation Inhibition

The ability of tRFs and tiRs to regulate global translation sets up a potential paradigm for designing a new class of small RNA therapeutics. tRF-5s and tiR-5s may be designed to inhibit translation in disease states where an aberrant upregulation in translation is essential for pathogenesis. For example, mRNA translation is an essential process in rapidly proliferating cancer cells (Malina et al., 2012). The mTOR inhibitor everolimus is used alone or as part of chemotherapeutics regimens in breast cancer, renal clear cell carcinoma, subependymal giant cell astrocytoma, and advanced neuroendocrine tumors (DRUGBANK, 2022). In the case of renal clear cell carcinoma, patients often develop resistance to mTOR inhibitors everolimus and temsirolimus (Voss et al., 2011). In the future, it will be interesting to evaluate the effectiveness of tRF-5/tiR-5 mediated global translation inhibition as a cancer therapy.

The benefit of tRF and tiR mediated translation inhibition as a therapeutic approach is that it does not require the target cell to contain or efficiently use the RNA interference machinery. This is especially important in the context of bacteria, which does not have a system analogous to RNA interference. Antimicrobial resistance is one of the top 10 global health threats to humanity according to the World Health Organization (WHO, 2022), therefore novel approaches are needed to address this issue. tRNA fragments were first described in *E. coli* (Levitz et al., 1990), but only recently has research into the global expression and biological roles of tRFs in microbes been conducted (Li and Stanton, 2021). Of note, several currently used antibiotics are small molecule inhibitors of translation (Chellat et al., 2016), positioning tRF and tiR mediated inhibition of translation as a viable approach. The development of antisense oligonucleotides as antibiotics is of great interest due to the ease of design, alterations in response to resistance, and clear targeting (Kole et al., 2012; Xue et al., 2018). Delivery of oligonucleotides is a grand challenge in eukaryotic oligonucleotide therapeutics (Hammond et al., 2021), and this challenge is no different in prokaryotes. However, conjugating oligonucleotides to peptides, vitamin B12, or encapsulation in nanoparticles has greatly improved the delivery of oligonucleotides into bacteria (Xue et al., 2018). It is also becoming clear that microbes (including Gram positive bacteria) are able to package RNAs and other cargo into extracellular vesicles (Domingues and Nielsen, 2017; Liu Y. et al., 2018; Toyofuku et al., 2019). These vesicles enable cross-talk between other bacteria as well as the host. As mammalian extracellular vesicles and other nanoparticles are being developed as vectors for carrying small RNA therapies (Bost et al., 2021), it is conceivable that bacterial vesicles may also function as drug delivery vehicles. This seems to be the case and has been an active area of study (Li and Liu, 2020). Most antibiotic oligonucleotide therapy development focuses on antisense technology, however, tRFs and tiRs that inhibit translation could also be conjugated or encapsulated into delivery vehicles and could provide another tool in the antibiotic oligonucleotide therapy toolbox.

Of note, one limitation of tRF/tiR antibiotics might be the role that tiRs appear to play in the regulation of the RNA repair operon (Hughes et al., 2020). The function of this operon is important for survival following DNA damage, and tiR-5s bind the CARF domain of RtcR, leading to oligomerization and activation of the RNA repair operon. Potentially giving a growth advantage to bacteria with DNA damage may be circumvented by utilizing tiR based therapies without a 3′ cyclic phosphate (3′ cP), as a 3′ cP was necessary for optimal CARF domain binding. The role of alternative 3′ ends in tRF/tiR mediated translation inhibition requires further study.

### Other Functions of tRFs

#### tRFs can Enhance Translation of Specific Transcripts

A surprising connection between tRFs and ribosomes was recently discovered. A 22 nucleotide tRF-3 derived from LeuCAG tRNA was found to bind and enhance the translation of RPS15 and RPS28 ribosomal mRNA (Kim et al., 2017). The tRF-3 binds RPS28 mRNA in the coding region across vertebrates and within the 3′ UTR in primates. The enhancement of translation is post-initiation and is conserved between mouse and human (Kim et al., 2019). Loss of this tRF results in apoptosis of cells in an orthotopic hepatocellular carcinoma model, indicating its importance in proliferation and tumorigenesis. These findings indicate that tRFs can be oncogenic.

#### tRFs can Bind Proteins and Affect Their Function

There are also reports of tRFs binding proteins and altering their function. For example, YBX1 binds to oncogenic transcripts and stabilizes them (Goodarzi et al., 2015). Internal tRFs (i-tRFs) derived from the anticodon region of parental tRNAs from Glu, Asp, Tyr, and Gly have a consensus sequence that binds and sequesters YBX1 from oncogenic transcripts, destabilizing them. These i-tRFs, therefore, function as tumor suppressors. A tumor suppressor tRF-3 derived from Glu tRNA was found to be expressed in normal mammary tissue but not breast cancer (Falconi et al., 2019). This tRF-3 could bind nucleolin (NCL), which sequestered NCL away from p53 mRNA and enhanced p53 mRNA translation (Falconi et al., 2019). In another study, 20 ANG dependent tiRs were found to interact with cytochrome c and reduce apoptosis in hyperosmotic conditions (Saikia et al., 2014). Finally, tRFs have also been implicated in viral pathogenesis. During tRNA maturation SSB/La binds to the trailer sequence and aids in tRNA maturation. Cho et al. (2019) found that SSB/La could interact with tRF_U3_1, derived from tRNA-Ser(TGA) also known as tRF-1001, and become sequestered in the cytoplasm. Since SSB/La binds to hepatitis C viral (HCV) internal ribosomal entry sites (IRES) (Pudi et al., 2003), sequestration of SSB/La by tRF_U3_1 reduced translation *via* the HCV IRES (Cho et al., 2019).

As we discover more biological roles of tRFs and tiRs and delineate their functions from miRNAs, we may begin to develop unique tRF-mimic and tRF antagonist based therapeutics. Such therapies are being developed for miRNAs, although there are no miRNA therapeutics in phase III clinical trials (Zhang et al., 2021). One major roadblock for miRNA based therapeutics that is absent in siRNA based therapeutics is the large number of putative miRNA targets, such that sponging or overexpression of miRNA may cause off-target effects (Zhang et al., 2021). This may also be a major roadblock in tRF based therapeutics, although rigorous prediction and validation of tRF targets is still in its infancy.

### Delivery of Oligonucleotide Therapeutics is a Major Challenge

Delivery of oligonucleotides to their site of action is a major challenge (Dowdy, 2017; Roberts et al., 2020; Hammond et al., 2021). tRF based therapeutics would have many of the same delivery issues. Oligonucleotide delivery can be broadly classified into two steps: 1) tissue delivery, and 2) cytoplasmic delivery or endosomal escape. Tissue specific delivery of oligonucleotides is more amenable to organs that are involved in blood filtration, such as the liver and kidney. Tissue delivery is also currently more amenable to organ systems that can be directly accessed *via* injection, such as the eye or brain and spinal cord by intrathecal injection. Furthermore, Alnylam has had success with liver specific delivery of siRNA-based therapies that are conjugated to N-acetylgalactosamine (GalNAc) (Springer and Dowdy, 2018). Lipid nanoparticles (LNPs) also enable more targeted delivery into tissues. LNPs have been successfully used for delivery of COVID-19 mRNA vaccines and the recently approved PCSK9 siRNA for the treatment of hypercholesterolemia (Fitzgerald et al., 2017; Buschmann et al., 2021).

Delivery of small RNAs to tissues may also be enhanced by encapsulation in extracellular vesicles (O’Brien et al., 2020). In support of the use of extracellular vesicles as tRF and tiR delivery agents, tRFs and tiRs are enriched in T cell extracellular vesicles (Chiou et al., 2018). tRFs were packaged into extracellular vesicles and released from activated T cells. Reduced expression of tRFs that are normally packaged and released leads to enhanced T cell activation. Although the effect of endogenous extracellular vesicle tRFs and tiRs on recipient cells is underexplored, it is clear that overexpressed tiRs can be delivered to recipient cells and alter gene expression (Gámbaro et al., 2020). Mouse epididymosomes can also deliver tRF-5s to maturing sperm, which ultimately represses MERVL (Sharma et al., 2016).

Another challenge for tissue delivery is oligonucleotide stability in circulation (Paunovska et al., 2022). Much of this issue has been solved, either by encapsulation of the oligonucleotide in an LNP, or by installment of RNA modifications (Yu et al., 2019). Interestingly, tRNAs, and likely tRFs, are one of the most highly modified RNAs in the cell, which may confer some enhanced stability in the circulation (Pan, 2018).

The second challenge of delivery, endosomal escape, is still a major unresolved bottleneck in the oligonucleotide therapeutics field (Dowdy, 2017). Small molecules such as nigericin and chloroquine have been found to enhance endosomal escape (Heath et al., 2019; Orellana et al., 2019), however, these compounds are too toxic for clinical use (Brown et al., 2020). It does appear that LNP platforms are better suited for endosomal escape than GalNAc conjugated siRNA mediated delivery, but this comes at the expense of duration of action (Brown et al., 2020). Any tRF or tiR therapeutic would also need to overcome these barriers.

### A Word of Caution for tRNA Based Therapeutics

Although tRNA based therapies are outside the scope of this review, it is a burgeoning field of development because nonsense suppressor tRNAs can prevent the formation of truncated, dysfunctional proteins (Porter et al., 2021). It is clear, however, that overexpressed tRNAs produce tRNA fragments. These tRNA fragments may cause off-target effects and lead to unpredictable side effects. In order to circumvent these effects, the biogenesis of tRFs and tiRs must continue to be thoroughly investigated. For example, it is clear that certain modifications can affect the production of tRFs ((He et al., 2021; Nagayoshi et al., 2021) and reviewed in (Lyons et al., 2018)). Parental tRNA cleavage induced by reactive oxygen species can also be prevented in T cells by binding Schlafen 2 (SLFN2) (Yue et al., 2021) (Figure 1). Findings like these will enable rational design of stable tRNA therapeutics.

## Conclusions and Future Directions

It is clear that tRFs/tiRs have important roles in biology in all domains of life. Mechanistically, tRFs/tiRs function differently, even between and within class distinctions. General trends in tRF/tiR’s role in regulating nascent gene silencing, post-transcriptional gene silencing, and mRNA translation make this class of small RNAs one of the most versatile classes discovered to date. Small RNA therapeutics based on basic knowledge about miRNA function are experiencing a renaissance, with several RNAi based therapeutics on the market. Perhaps tRFs and tiR based therapeutics will follow suit. In order for tRFs/tiRs to reach the clinic, several challenges must be addressed. For example, it is clear that tRF-5s and tiR-5s can behave as protein synthesis inhibitors. What is less clear is whether there is an RNA modification or RNA sequence code that is important for tRF-5 and tiR-5 mediated protein synthesis inhibition. For example, PUS7 mediated pseudouridylation is important for protein synthesis inhibition in stem cells (Guzzi et al., 2018; Guzzi et al., 2022). Since tRNAs have on average thirteen modifications per molecule (Pan, 2018), it would not be surprising if other tRF/tiR modifications regulate protein synthesis, perhaps in a combinatorial or tissue specific manner. tRF/tiR modifications may provide other useful roles and insights into tRF and tiR based therapeutics. For example, tRF and tiR modifications from species adapted to extreme conditions, such as some bacteria and archea (Babski et al., 2014; Li and Stanton, 2021), may be useful for further stabilization of synthetic tRFs and tiRs or confer novel functions.

Another challenge is understanding the role that tRFs play in nascent RNA silencing and post-transcriptional gene silencing. Do all tRFs follow the same rules (i.e. seed based pairing, supplemental base pairing, etc) as miRNAs when it comes to post-transcriptional gene silencing? What role do tRF modifications play in nascent RNA silencing and post-transcriptional gene silencing? Since most tRF target prediction tools are built on miRNA-based assumptions and rules or more general complementary pairing (Li et al., 2021; Xiao et al., 2021; Zhou et al., 2021), are we capturing the most robust tRF targets? Finally, it is unclear what features of tRFs/tiRs make them more likely to behave primarily as miRNAs, interact with RNA binding proteins, or interact with other RNAs. In order for tRFs/tiRs to be useful in the clinical setting, these questions will need to be addressed.
